# Breakthrough Seizures in Children Receiving Valproate Therapy

**DOI:** 10.7759/cureus.86273

**Published:** 2025-06-18

**Authors:** Arunima Gupta, Anju Aggarwal, Sangeeta Sharma, Aaradhana Aaradhana

**Affiliations:** 1 Pediatrics, University College of Medical Sciences and Guru Tegh Bahadur Hospital, Delhi, IND; 2 Neuropsychopharmacology, Institute of Human Behaviour and Allied Sciences, Delhi, IND

**Keywords:** breakthrough seizures, children, epilepsy, therapeutic drug monitoring, valproate

## Abstract

Introduction

Breakthrough seizures in children diagnosed with epilepsy pose a significant clinical challenge. Though valproate is effective in managing seizures, a subset of patients can still experience breakthrough seizures. It can cause increased morbidity, reduce the quality of life, and increase anxiety among parents. There is insufficient data in the Indian population regarding the cause of breakthrough seizures and their association with serum valproate levels; hence, we carried out this study.

Objective

Primary Objective

To study the clinical profile and drug levels in children presenting with breakthrough seizures while receiving valproate therapy.

Secondary Objective

To find out the association of the clinical, demographic, and etiological profile of breakthrough seizures with valproate levels.

Methods

A group of 100 children, 2-12 years old, receiving valproate therapy and presenting to the hospital within 24 hours of a breakthrough seizure were studied. Clinical and demographic profiles were recorded. Valproate levels were estimated using recombinant DNA technology using an autoanalyzer (reference level 50-100 mcg/ml). Phenytoin levels were estimated in 10 children receiving dual therapy (reference level 10-20 mcg/mL).

Results

The mean age was 81.6±31.9 months (59 M, 41 F). The mean dose of valproate was 24.8±8.4 mg. Syrup formulation was used in 73 and tablets in 27. Non-compliance was observed in 19, and fever was identified as a possible precipitating factor in 18. Compliance was not affected by age, sex, socioeconomic status, education of parents, or type of seizure (p > 0.05). Valproate levels were below the reference range in 44, within the reference range in 47, and above the reference range in 9. There was no statistically significant association between valproate levels (therapeutic or subtherapeutic) and age, sex, socioeconomic status, parental education, seizure type, or the presence of precipitating factors (p > 0.05). Similarly, mean valproate levels were also not affected by these factors. The levels did not vary with drug formulations.

Conclusion

Breakthrough seizures in children on valproate therapy were not related to valproate levels. Valproate levels were not affected by clinical or demographic profile, drug formulation, change in formulation, compliance, etc.

## Introduction

Epilepsy is a chronic neurological condition of the brain, impacting approximately 50 million individuals globally, according to WHO [1]. Antiseizure medications (ASMs) stand as the foundation of epilepsy treatment. The principal aim in managing epilepsy is to attain seizure control using optimal doses of ASM while avoiding and averting any potential toxicity. Valproate is a broad-spectrum antiseizure medication and is the treatment of choice for generalized tonic-clonic and myoclonic seizures [2]. Though ASMs are effective in managing seizures, a subset of patients can still experience breakthrough seizures, which are epileptic episodes that occur despite the use of antiepileptic drugs that have otherwise successfully prevented seizures in the patient [3]. According to estimates, 60 to 70% of patients with epilepsy will experience a seizure remission. However, up to 37% of them can have a breakthrough seizure [4]. It can cause increased morbidity, reduce the quality of life, and increase anxiety among parents. Seizure control may be affected by several factors, including drug formulation, drug levels, and poor compliance. Various factors like fever, infections, watching television, sleep deprivation, etc., have been observed to precipitate seizures in children [5]. Measuring serum drug levels, called therapeutic drug monitoring, seeks to enhance clinical outcomes, prevent adverse effects, and minimize the cost associated with drug therapy [6].

There is insufficient data regarding the association of breakthrough seizures with changes in drug formulation, other precipitating factors, and drug levels below which breakthrough seizures are precipitated. Hence, this study was planned to find out the association between breakthrough seizures with drug levels and the clinical profile of patients.

## Materials and methods

This cross-sectional study was carried out over 18 months in a tertiary care hospital in Delhi from September 2022 to February 2024. Ethical clearance was obtained from the institutional ethics committee (IECHR-2022-55-38), and written informed consent was obtained from the parents/guardians.

Selection and description of participants

Children between the ages of 2 and 12 years who were receiving treatment with valproate alone or in combination with another ASM (phenytoin) for at least three months were included in the study. These children presented to the emergency or outpatient department with breakthrough seizures. They were either in active seizure or had a seizure episode in the past 24 hours. Children who had received a loading dose of any anti-seizure medication prior to presentation, as well as those with known chronic liver disease, chronic renal disease, syndromic epilepsy, or clinical meningitis or encephalitis, were excluded from the study. According to a previous study, the proportion of children receiving valproate and presenting with breakthrough seizures and having ASM levels below the reference range varied from 34.2% to 44.1% [7]. Expecting 45% in the present study, with 25% relative precision on either side and with a 95% confidence level with continued correction, a sample size of 84 was found to be sufficient. So, 100 subjects were enrolled for the present study. A wider precision was chosen to ensure a feasible sample size within the constraints of the study’s single-center design, timeframe, and available resources.

Data collection

Information regarding patients’ demographic profiles, clinical history, compliance, seizure characteristics, medication formulation, method of drug measurement, and relevant investigations was recorded in a pre-designed case record form to aid in the identification of the type of seizure and associated risk factors.

Children presenting with an active seizure were initially treated with IV midazolam at the dose of 0.1 mg/kg, followed by further management of the seizure disorder as per the hospital’s protocol. A 3 ml venous blood sample was collected before administering the loading dose of anti-seizure medication. The serum was separated within 30 minutes stored at 0-8°C and was processed within 24 hours of collection. Phenytoin levels were also measured if the children were on phenytoin alongside valproate. Drug levels were estimated using CEDIA II kits (Thermofisher Scientific/2018), which use recombinant DNA technology to develop a uniform immunoassay system. The minimum detectable concentration of CEDIA Valproic Acid II and Phenytoin II assays was 3.0 mcg/ml and 0.6 mcg/ml, respectively. The laboratory reference range for valproate was taken as 50 to 100 mcg/mL, and for phenytoin, it was taken as 10 to 20 mcg/mL as per laboratory and kit specifications. The study aimed to investigate whether valproate levels below the reference range were associated with breakthrough seizures.

Statistical analysis

Data analysis was performed using IBM Corp. Released 2012. IBM SPSS Statistics for Windows, Version 20.0. Armonk, NY: IBM Corp. Chi-square/Fisher exact tests were used to determine the association between drug levels being in the therapeutic or subtherapeutic range and the clinical and demographic profile of children with breakthrough seizures. Nonparametric Mann-Whitney and Kruskal-Wallis tests were applied to assess the effect of clinical and demographic profiles on mean and median valproate levels. Since the number of children having valproate levels above the reference range (>100 mcg/ml) was less, and we wanted to assess whether valproate levels below the reference range (<50 mcg/ml) were a cause of breakthrough seizures, children with valproate levels less than the reference range (<50 range(<50mcg/ml) v/s the rest (≥50 mcg/ml) were compared. Multivariate analysis was performed using logistic regression.

## Results

A total of 100 children (59 males, 41 females) were enrolled with a mean age of 81.57±31.94 months. Out of the 28 children aged less than five years, 21.4% were severely stunted (height for age <85% expected), and 17.8% were severely wasted (weight for height <70% expected), according to WHO classification for malnutrition. Of children more than five years old, 20.8% had short stature. Microcephaly was seen in 19 children. Developmental delay was found in 59 children, and 31 had cerebral palsy. Generalized seizures were seen in 68 children, out of which 41 had tonic-clonic seizures and 27 had tonic seizures. None of the children had myoclonic or atonic seizures. Out of the 32 children who experienced focal seizures, 13 children had secondary generalization, 6 children had impaired awareness, and the rest, 13 children, had focal seizures without impaired awareness. Seizure types were classified according to the ILAE 2017 classification, based on caregiver-reported semiology and clinical observation. Fever was found to be a possible precipitating factor in 18 children. In the study group, 38 children had a history of previous breakthrough seizures, indicating poor seizure control. Noncompliance was observed in 19 children, and 13 children were receiving valproate in a dose that is considered subtherapeutic. Valproate was administered in tablet form to 27 children and as syrup to 73. Exact methods of measuring drug volume for administration were used in 82 children, which included syringes, calibrated containers, and tablet formulation. Phenytoin was prescribed along with valproate in 10 children. Neuroimaging was performed for all children and was normal in 47. Baseline laboratory parameters, including liver and kidney function tests and serum albumin, were recorded and were within the normal range in all children, ensuring that they did not have seizures due to electrolyte imbalance or hypoglycemia.

Valproate levels were below the reference range in 44 children (95% CI: 34.1 to 54.3%), in the reference range in 47 children, and above the reference range in 9 children (Table 1). In 11 children, valproate levels were below the detection level, and for calculation purposes, a level of 3.0 mcg/mL was considered, which is the minimum detectable concentration according to the CEDIA Valproic Acid II assay. Phenytoin levels were found to be below the reference range in all 10 children and below the detection range in four of them. For calculation, a level of 0.6 mcg/ml was considered, which is the minimal detectable concentration (Table 1).

**Table 1:** Anti-seizure medication levels in children with breakthrough seizures

Valproate Levels (mcg/mL)	Number of Children (n=100)	Phenytoin Levels (mcg/mL)	Number of Children (n=10)
Below Reference Range (<50mcg/mL)	44	Below Reference Range (<10mcg/mL)	10
In the Reference Range (50-100mcg/mL)	47	In the Reference Range (10-20mcg/mL)	0
Above Reference Range (>100mcg/mL)	9	Above Reference Range (>20mcg/mL)	0

When we studied the effect of age, sex, socioeconomic status, parent’s education, type of seizure, and the presence or absence of precipitating factors on whether valproate levels fell in the therapeutic or subtherapeutic range, no statistically significant difference was found (p-value of > 0.05) (Table 2). Similarly, mean valproate levels were also not affected by these factors. The levels did not vary with drug formulations (p-value of > 0.05).

**Table 2:** Effect of demographic and clinical profile on valproate levels Pearson’s chi-square test. *Fisher’s exact test. p-value of < 0.05 = significant

Parameters		Valproate level <50mcg/ml (n=44)	Valproate level ≥50mcg/ml (n=56)	p-value	Test Value
Sex	Male	26	33	0.987*	0.000*
	Female	18	23		
Age	24-60 months	12	16	0.974*	0.096*
	61-96 months	21	25		
	97-144 months	11	15		
Mother's education	Illiterate/Primary	20	23	0.155**	3.993**
	Middle/High/Intermediate	24	28		
	Graduate/Postgraduate	0	5		
Father's education	Illiterate/Primary	13	13	0.602**	1.034**
	Middle/High/Intermediate	29	38		
	Graduate/Postgraduate	2	5		
Socioeconomic status	Upper/Upper Middle/Lower Middle	27	41	0.207*	1.590*
	Upper Lower/Lower	17	15		
Drug formulation	Syrup	31	42	0.611*	0.258*
	Tablet	13	14		
Type of seizure	Generalized	31	37	0.641*	0.218*
	Focal	13	19		
Compliant with medication	Yes	35	46	0.742*	0.108*
	No	9	10		
Dose of valproate taken by the patient	<20mg/kg/day	9	4	0.049* (significant)	3.861*
	≥20/kg/day	35	52		
Developmental delay	Yes	19	22	0.694*	0.155*
	No	25	34		

Compliance also was not affected by age, sex, socio-economic status, education of parents, and type of seizure (p-value of > 0.05) (Table 3).

**Table 3:** Effect of demographic and clinical profile on compliance *Pearson’s chi-square test. **Fisher’s exact test. p-value of <0.05 = significant

Parameters		Compliant (n=81)	Non-complaint (n=19)	p-value	Test value
Sex	Male	46	13	0.354*	0.861*
	Female	35	6		
Age	24-60 months	23	5	0.465**	1.571**
	61-96 months	35	11		
	97-144 months	23	3		
Mother's education	Illiterate/Primary	32	11	0.343**	2.191**
	Middle/High/Intermediate	44	8		
	Graduate/Postgraduate	5	0		
Father's education	Illiterate/Primary	18	8	0.206**	3.039**
	Middle/High/Intermediate	57	10		
	Graduate/Postgraduate	6	1		
Socioeconomic status	Upper/Upper Middle/Lower Middle	55	13	0.965*	0.002*
	Upper Lower/Lower	26	6		
Drug formulation	Syrup	60	13	0.617*	0.250*
	Tablet	21	6		
Type of seizure	Generalized	57	8	0.294*	1.101*
	Focal	24	11		
Developmental delay	Yes	49	10	0.531*	0.3938*
	No	32	9		

## Discussion

This prospective study was done to assess the association of breakthrough seizures with the clinical profile and anti-epileptic drug levels, specifically valproate, in children aged 2 to 12 years receiving valproate therapy. Among the 100 children enrolled, 90 were on monotherapy with valproate, while 10 received a combination of valproate and phenytoin.

The serum valproate levels varied widely. Forty-four children had levels below the reference range (<50 mcg/ml), 47 within the reference range (50-100 mcg/ml), and only 9 had levels above 100 mcg/ml. These findings are similar to prior Indian studies, including those by Garg et al. [7], Harivenkatesh et al. [3], and Kumar et al. [5], which also reported a substantial proportion of pediatric patients with subtherapeutic valproate levels despite treatment. The mean valproate level in this study was 57.49±33.83 mcg/ml.

All 10 children receiving phenytoin had levels below the reference range. This is similar to a study by Garg et al. [7]. In contrast, in another Indian study [3], only 43.13% of children were found to have phenytoin levels below the reference range. Such low levels of phenytoin may be explained by genetic polymorphisms leading to metabolic variability, the narrow therapeutic index of phenytoin, and inadequate shaking of the drug suspension before use leading to inconsistent drug concentrations [8].

Multiple contributory factors for breakthrough seizures could be identified, like fever, noncompliance planned tapering of drug dose, prior poor seizure control, and low dose taken by the patient. However, none of them had a statistically significant effect on the valproate levels except the inappropriately low dose taken by the patients (p-value of 0.049) (Table 2). Multivariate analysis was performed, and no statistically significant association was found among these factors. Common precipitating factors observed in studies by Fang PC [9] and Kumar S [5] were fever, emotional stress, sleep deprivation, and watching television.

Non-compliance was observed in 19 children, the most common reason being forgetfulness, seen in 13 (68.4%) children, medication unavailability in five children, and discontinuation by the parent without medical consultation in one case. This is similar to a study by Aldosari AN et al. [10], in which 34.3% of subjects were found to be non-compliant, with 67.5% citing forgetfulness as the most common cause. However, compliance did not significantly affect the serum drug levels in this study (p-value of 0.742). This contrasts with the findings of Charfi et al. [11], where compliance had a significant impact on achieving therapeutic drug levels. The difference in the findings may be due to variations in adherence assessment methods or a possible response bias.

Socioeconomic status and parents' educational background can impact their access to healthcare and understanding of the medical condition, thereby indirectly affecting compliance. No statistically significant association was found between parents’ literacy and compliance with medication or drug levels in this study. This is in contrast to an interview-based study done by Paschal AM [12], in which it was found that higher health literacy and parental education were associated with better compliance and fewer missed drug doses. This can suggest that in resource-limited settings, other systemic or behavioral factors may play a bigger role than just parents’ education or income.

Children with developmental delay and cerebral palsy are at a higher risk of breakthrough seizures due to underlying brain deformities [13]. This study found no significant association between these conditions and serum valproate levels (p-value of >0.05). Mean valproate levels were 64.03±34.18 mcg/ml in children with cerebral palsy and 54.55±33.50 mcg/ml in those without cerebral palsy. Although not statistically significant, the higher mean drug levels in children with cerebral palsy may be due to higher prescribed doses for recurrent seizures and better medication compliance by the parents. However, genetic and metabolic testing could not be routinely performed in this study, which might limit the ability to distinguish pharmacoresistance from subtherapeutic drug exposure. Future studies with syndromic classification and genetic profiling may help to identify children with inherent treatment resistance.

It was observed that 56 children presenting with breakthrough seizures had valproate levels ≥50 mcg/ml, which might be explained by variable pharmacokinetics of the drug and variation in drug metabolism among children. This highlights that having a therapeutic drug level does not always ensure seizure control. Thus, as suggested by ILAE, the concept of individual therapeutic concentrations should be taken into consideration, as the reference ranges are not tailored to specific age groups and are generalized across all ages [14]. Supporting this, a randomized controlled trial by Jannuzzi et al. [15] showed no significant difference in the 12-month remission rates between patients whose ASM doses were adjusted based on therapeutic drug monitoring (TDM) and those adjusted on clinical grounds (60% vs. 61%). This suggests that clinical judgment may be as effective and further emphasizes the need for individualized treatment approaches.

The advantages of this study were that this study focused on a homogeneous group of children receiving predominantly a single ASM, valproate. It was a prospective study. In contrast, most previous studies were retrospective in nature relied on laboratory data, and included a heterogenous population of both adults and children, sometimes involving multiple ASMs. The main limitation of this study was that samples for drug level estimation were collected within 24 hours of a breakthrough seizure, rather than at the time of seizure; there was no longitudinal follow-up of the patients, and there was a limited sample size.

This study concludes that there is no statistically significant association between breakthrough seizures and serum valproate levels, compliance with medication, seizure type, or clinical profile. The only variable significantly associated with valproate levels was the prescribed dose, emphasizing the importance of correct dosing. Breakthrough seizures were observed in many children with valproate levels in the reference range, indicating that serum drug levels might not be the only variable predicting seizure control in children. Studies with a larger number of children and serial drug monitoring may be required for conclusive results.

## Conclusions

This study investigated the association between breakthrough seizures and serum valproate levels in children receiving valproate therapy. A significant proportion of children with breakthrough seizures had valproate levels within the reference range, suggesting that factors other than drug levels, such as individual pharmacokinetics, genetic variability in drug metabolism, and patient-specific therapeutic thresholds, may influence seizure control. No significant association was found between serum valproate levels and age, gender, type of seizure, presence of fever, medication compliance, change in drug formulation, parental education, socioeconomic status, or coexisting conditions like developmental delay and cerebral palsy. Overall, the study concludes that breakthrough seizures in children on valproate therapy cannot be reliably predicted by serum drug levels or conventional clinical parameters, highlighting the need for individualized therapeutic approaches because of the complex nature of seizure management in children.
